# Neutralizing Antibodies in Patients with Chronic Hepatitis C, Genotype 1, against a Panel of Genotype 1 Culture Viruses: Lack of Correlation to Treatment Outcome

**DOI:** 10.1371/journal.pone.0062674

**Published:** 2013-05-07

**Authors:** Jannie Pedersen, Tanja B. Jensen, Thomas H. R. Carlsen, Kristian Schønning, Peer Brehm Christensen, Alex Lund Laursen, Henrik Krarup, Jens Bukh, Nina Weis

**Affiliations:** 1 Department of Infectious Diseases, Copenhagen University Hospital, Hvidovre, Denmark; 2 Copenhagen Hepatitis C Program (CO-HEP), Department of Infectious Diseases and Clinical Research Centre, Copenhagen University Hospital, Hvidovre, Denmark; 3 Department of Clinical Microbiology, Copenhagen University Hospital, Hvidovre, Denmark; 4 Department of Infectious Diseases, Odense University Hospital, Odense, Denmark; 5 Department of Infectious Diseases, Aarhus University Hospital, Skejby, Denmark; 6 Department of Clinical Biochemistry, Aalborg University Hospital, Aalborg, Denmark; 7 Faculty of Health and Medical Sciences, University of Copenhagen, Copenhagen, Denmark; 8 Department of International Health, Immunology and Microbiology, Faculty of Health and Medical Sciences, University of Copenhagen, Copenhagen, Denmark; Inserm, U1052, UMR 5286, France

## Abstract

The correlation of neutralizing antibodies to treatment outcome in patients with chronic hepatitis C virus (HCV) infection has not been established. The aim of this study was to determine whether neutralizing antibodies could be used as an outcome predictor in patients with chronic HCV, genotype 1, infection treated with pegylated interferon-α and ribavirin. Thirty-nine patients with chronic hepatitis C, genotype 1a or 1b, with either sustained virologic response (n = 23) or non-sustained virologic response (n = 16) were enrolled. Samples taken prior to treatment were tested for their ability to neutralize 6 different HCV genotype 1 cell culture recombinants (1a: H77/JFH1, TN/JFH1, DH6/JFH1; 1b: J4/JFH1, DH1/JFH1, DH5/JFH1). The results were expressed as the highest dilution yielding 50% neutralization (NAb_50_-titer). We observed no genotype or subtype specific differences in NAb_50_-titers between patients with chronic HCV infection with and without sustained virologic response when tested against any of the included culture viruses. However, NAb_50_-titers varied significantly with a mean reciprocal NAb_50_-titer of 800 (range: 100–6400) against DH6/JFH1 compared to a mean NAb_50_-titer of 50 (range: <50–400) against all other included isolates. Subsequent studies demonstrated that the efficient neutralization of DH6/JFH1 could be linked to engineered adaptive mutations in the envelope-2 protein. In analysis of envelope 1 and 2 sequences of HCV, recovered from a subset of patients, we observed no apparent link between relatedness of patient sequences with culture viruses used and the corresponding neutralization results. In conclusion, pre-treatment levels of neutralizing antibodies against HCV genotype 1 isolates could not predict treatment outcome in patients with chronic HCV infection. High neutralization susceptibility of DH6/JFH1 could be correlated with adaptive envelope mutations previously highlighted as important for neutralization. Our study emphasizes the importance of using multiple culture viruses for neutralization studies and contributes to the current knowledge about neutralizing epitopes, important for future therapeutic- and vaccine-studies.

## Introduction

Hepatitis C virus (HCV) is a human pathogen infecting approximately 170 million people worldwide, hereby increasing the risk of developing serious chronic liver diseases including liver cirrhosis and hepatocellular carcinoma (HCC) [1].

The standard of care treatment for HCV genotype 1 infected patients has for the last decade been a combination therapy of pegylated interferon-α and ribavirin (PEGIFN/RBV) for 48 weeks [2]. The effect of this treatment regimen is monitored, by measuring the HCV RNA levels in serial blood samples. Only about 50% of the treated patients achieve a Sustained Virologic Response (SVR), defined as undetectable HCV RNA 24 weeks post treatment termination. Early Virologic Response (EVR) is defined as negative or ≥2 log_10_ decrease in HCV RNA 12 weeks after treatment initiation. Patients with EVR are more likely to achieve SVR, while patients without EVR have a significant reduced chance of SVR and therefore will terminate treatment at this point of time [3].

In 2011, two promising NS3/4A protease inhibitors were introduced as an add-on to the PEGIFN/RBV treatment, improving the response rate to approximately 70% [4]–[6]. Unfortunately, the 3-drug therapy also increases the number of adverse events, and severe skin reactions like Drug Reaction with Eosinophilia and Systemic Symptoms (DRESS) and Steven Johnson syndrome have been reported [4], [7]. This indicates the continued need for predictive factors, enabling clinicians to evaluate the most likely treatment outcome for their patients.

Several host-, viral-, and therapeutic- factors have been reported as predictors of outcome of combination treatment with PEGIFN/RBV [2], [8]–[11]. As independent factors, genotype, baseline viral load, age at treatment initiation, IL28β genotype, IP-10 level, and duration of treatment have consistently been found to be strong predictors [2], [9], [12]. A systematic approach, regarding possible predictive factors in relation to the viral life cycle, has until recently been hampered by the lack of robust cell-culture systems. However, in 2005 a cell-culture system, based on HCV strain JFH1 from a Japanese patient, was developed [13]. The subsequent development of JFH1-based virus systems, expressing strain specific envelope proteins, permitted cross genotype *in vitro* neutralization studies [14]–[21]. These systems function as important tools, studying the complete viral life cycle and factors with influence on virus fitness like neutralizing antibodies and host cell factors. Various studies have shown that a broad and vigorous cellular immune response is needed to clear the virus in the acute phase [22], [23] where the role of NAb is less clear [24]–[27]. In the chronic infection, defined as HCV viremia persistence more than 6 months, the virus persists despite HCV specific T-cell responses. In most of these patients high levels of NAb can be detected. It has been suggested that a continued pressure from NAbs during this stage drives viral evolution and reduces viral load compared to the acute stage, and a recent study showed that an initial strong NAb response were able to facilitate a subsequent efficient cellular immune response leading to clearance of a chronic infection [22], [27]–[29]. As it has been shown that a high viral load has a negative effect of PEGIFN/RBV treatment [30], one could speculate that a high level of NAb prior to treatment could lower the viral load and thereby increase the chances of treatment-induced viral clearance.

Only two studies have looked at the predictive value of NAb on treatment effect but with contradictive results [31], [32]. Thus, there is a need for further investigation in this research area. The aim of the present study was to investigate whether the presence of NAb influences the outcome of PEGIFN/RBV treatment in patients with chronic HCV, genotype 1, infection. The patient plasma was tested against six genotype 1 recombinant cell culture viruses in a neutralizing assay. We found no significant difference between patients with and without a SVR, but interestingly the study revealed significant differences in NAb-titer between the culture viruses. These differences were subsequently linked to the presence of adaptive mutations in epitopes previously found critical for neutralization.

## Materials and Methods

### Patient Selection and Sample Collection

The patients were selected through the Danish Database for Hepatitis B and C [33], [34].

Inclusion criteria:

1. Patients with chronic hepatitis C, genotype 1, who had been treated with PEGIFN/RBV for a minimum of 12 weeks.
2. Available pre-treatment sample in the DANHEP biobank.
3. Known treatment outcome; sustained virologic response (SVR) or non-sustained virologic response (non-SVR), corresponding to either non-detection or detection of HCV-RNA 24 weeks after end of treatment.
4. Patients treated at Aalborg University Hospital, Odense University Hospital, Copenhagen University Hospital, Hvidovre, or Aarhus University Hospital, Skejby.

Exclusion criteria:

1. Patients co-infected with hepatitis B virus (HBV) or human immunodeficiency virus (HIV).
2. Patients who discontinued treatment due to lack of compliance or adverse events.
3. Patients with chronic hepatitis C, genotypes non-1, infection.

From the 4 different hospitals in Denmark involved in this study, only 39 (SVR = 23, non-SVR = 16) met the criteria; 10 patients from Aalborg University Hospital, 14 patients from Odense University Hospital, 6 patients from Copenhagen University Hospital, Hvidovre, and 9 patients from Aarhus University Hospital, Skejby. This did not meet the number of 31 patients in each group, as calculated prior to study initiation to be able to detect a difference of 150 in the reciprocal neutralization titer, defined as the highest dilution at which the plasma neutralized 50% of the virus (NAb_50_-titer) with a power of 80% and a 0.05 level of significance. However, all patients who fulfilled the criteria were included.

The patients were divided into two groups, according to treatment outcome (SVR and non-SVR) and compared at baseline (Table 1). 90% of the patients in both groups had the sample taken within a year prior to treatment initiation. The remaining patients had the sample taken within 2.5 years prior to treatment. All patients were treated with a weekly dose of 80–180 µg pegylated interferon-α 2a or 2b in combination with 800–1400 mg ribavirin daily.

**Table 1:** Baseline data of 39 patients chronically infected with HCV, genotype 1, divided in groups with either SVR or non-SVR following pegylated interferon/ribavirin treatment.

	SVR	Non-SVR
Total	23	16
1a	14 (61%)	12 (75%)
1b	9 (39%)	3 (19%)
Not determined	0 (0%)	1 (6%)
Male	16 (70%)	11 (69%)
Female	7 (30%)	5 (31%)
Intravenous drug users (IDU)	6 (26%)	8 (50%)
Non-IDU	6 (26%)	5 (31%)
Unknown	11 (48%)	3 (18%)
European	19 (83%)	14 (88%)
Non-European	4 (17%)	2 (12%)
BMI >25	10 (43%)	8 (50%)
BMI ≤25	10 (43%)	5 (31%)
Unknown	3 (13%)	3 (19%)
Biopsy yes	16 (70%)	10 (63%)
Biopsy no	7 (30%)	6 (37%)
Cirrhosis yes*	3 (19%)	6 (60%)
Cirrhosis no*	13 (81%)	4 (40%)
Age ≤45	8 (34%)	3 (19%)
Age >45	15 (65%)	13 (81%)
HCV RNA ≥600.000	16 (70%)	10 (63%)
HCV RNA <600.000	7 (30%)	6 (37%)
ALT ≥70/100	13 (57%)	8 (50%)
ALT <70/100	10 (43%)	8 (50%)
Treatment duration ≥48 weeks	18 (78%)	6 (37%)
Treatment duration <48 weeks	5 (22%)	10 (63%)

The data is shown in absolute numbers and percent of the total in the group. None of the baseline data was significant predictors of pegylated interferon/ribavirin treatment outcome in this cohort.

* only patients with a biopsy were included. SVR = sustained virologic response, non-SVR = non sustained virologic response.

### Subtyping

HCV RNA was extracted from patient plasma samples using an automated in-house procedure. One-step RT-PCR was conducted using Core/E1 specific primers DM110 and DM111 as described [11], [35] and yielded amplicons for all individuals except one. Amplicons were sequenced directly (Macrogen). Core-E1 sequences (nts. 868–1288 (reference strain H77, GenBank accession number AF009606)) of the 38 patients, the six included genotype 1 culture viruses, and culture viruses of the other major genotypes with genotype 7a (QC69) as an out-group were aligned using ClustalW. The alignment was manually rearranged to obtain codon-based nucleotide alignment. Phylogenetic tree was generated using the Jukes Cantor model in Neighbor-joining algorithm. Bootstrap 1000 values indicate similarities of 1000 trees in percentage. Reference sequences used for genotype assignment were obtained from the Los Alamos HCV Database Project.

### Determination of Patient Derived HCV Envelope 1 and 2 Sequences

The HCV E1E2 sequence from 16 patient derived viruses (8 genotype 1a and 8 genotype 1b) was determined. HCV RNA was extracted from 100–200 µL plasma samples using High Pure Viral Nucleic Acid kit from Roche or from 50 µL using Trizol Reagent from Invitrogen. Reverse transcription PCR (RT-PCR) was done using SuperscriptIII (Invitrogen) and RT-primer OREV-CE1E2 (5′ TGGTGGTAACGCCAGCAGGA3′) [36] for the genotype 1b patient samples and 2826R_GT1a (5′ GCCATTAACCCGACAAGAAC3′) for the genotype 1a patient samples. For PCR, the Advantage 2 PCR Enzyme System was used with the reverse primers described above and the forward primer OFOR-CE1E2 (5′ AAYTTGGGTAAGGTCATCGATACC3′) [36]. The PCR was run in 36 cycles of 95°C for 35 sec, 55°C for 30 sec, and 68°C for 7 min. For the nested PCR IFOR-CE1E2 (5′ CGCCGACCTCATGGGGTACATT3′) [36] and 2661R_GT1a (5′ GCAGAAGAACACGAGGAAGG3′) were used for the genotype 1a patient samples and IFOR-CE1E2 (listed above) and IREV-CE1E2 (5′ GAAGAACACAAGGARGGAGAG3′) [36] or 2756R_GT1b (5′ CCAGCAGGAGCAGGAGCAGC3′) for the genotype 1b patient samples, using the same PCR cycling parameters. Amplicons were sequenced directly (Macrogen). The patient derived sequences of E1E2 (nts 915–2579 (H77, GenBank accession number AF009606)) were aligned with the corresponding sequence of the six included genotype 1a and 1b culture viruses and compared in a phylogenetic analysis as described above.

### Recombinant Viruses

The 39 plasma samples included were tested against six recombinant viruses: Three genotype 1a (H77/JFH1, TN/JFH1, and DH6/JFH1) and three genotype 1b (DH1/JFH1, DH5/JFH1, and J4/JFH1). All viruses were generated prior to this project [14], [16], [19]. The recombinant viruses contain the Core-NS2 strain specific sequence, determined by a consensus sequence and incorporated into the JFH1 recombinant. All included recombinant virus infected the majority of Huh7.5 cells in culture with peak infectivity titers of 10^3.2^–10^4.3^ focus forming units (FFU)/mL. Robust infection with each recombinant required the presence of adaptive mutations. The following adapted recombinants were used in the present study: H77/JFH1_V787A,Q1247L_, DH6/JFH1_V157A, I414T, Y444H, V787A, S905C Q1247L_, TN/JFH1_R1408W,_ J4/JFH1_F886L,Q1496L_, DH1/JFH1_F886L, Q1496L_, and DH5/JFH1_C734W, F886L, R1369L, Q1496L_. The E1E2-sequence of the included culture viruses is shown in Figure S1. In the remainder of the manuscript the culture viruses are listed without adaptive mutations. For subsequent analysis DH6/JFH1_V157A, V787A, S905C, Q1247L_ was used. This construct will be listed with the adaptive mutations.

Virus stocks were previously made for J4/JFH1 [14]. New virus stocks were made for H77/JFH1 (3^rd^ viral passage), TN/JFH1 (2^nd^ viral passage), DH6/JFH1 (2^nd^ viral passage), DH1/JFH1 (2^nd^ viral passage), DH5/JFH1 (2^nd^ viral passage), and DH6/JFH1_V157A, V787A, S905C, Q1247L_ (2^nd^ viral passage) by inoculating Huh7.5 cells with a multiplicity of infection (MOI) of approximately 0.003. Supernatants were harvested and the cultures closed at estimated infection of 80–90% of the cells (day 9–12). HCV envelope sequences of recovered viruses were verified by direct sequencing to confirm their authenticity and to exclude further adaptive mutations. The procedure is described in greater detail elsewhere [14], [20].

### Neutralization Assay

The titer of neutralizing antibodies was investigated using the HCV cell culture (HCVcc) system with the above-mentioned viruses. All plasma samples were blinded prior to experiment initiation and each patient were given a study number unrelated to the patient’s personal data.

For the neutralization test [14]–[16], 30–150 FFU of the included recombinant viruses were incubated for 1h with 2-fold dilutions of heat-inactivated (56°C for 30 min) plasma in three replicates. This was followed by 3 h of incubation on 6×10^3^ Huh7.5 cells/well in poly-D-lysine-coated-96 well plate (Nunc) before washing and incubation for 48 h in complete medium. The cells were immunostained for HCV NS5A (using the NS5A specific antibody 9E10) [18]. Secondary antibody was ECL anti-mouse IgG horseradish peroxidase-linked whole antibody (GE Healthcare Amersham, Buckinghamshire, United Kingdom) and staining obtained using a DAB+ Substrate Chromogen System (DAKO, Glostrup, Denmark). The number of FFU was determined by using ImmunoSpot Series 5 UV Analyzer (CTL Europe GmbH, Bonn, Germany) with customized software [37]. The mean background level of 6 negative wells was below 8 in all experiments; the negative mean was subtracted from FFU counts in experimental wells. Percent neutralization was calculated by relating the mean FFU of the experimental wells in three replicates to the mean of six replicate cultures inoculated with virus only as previously described [15], [16], [37], and the results was given in NAb_50_-titer. The lowest dilution used in the present study was 1/50.

As controls, plasma and serum from the same patient taken at the same time point were tested simultaneously against DH6/JFH1 to test for assay variation between the two materials; two different patients, one with SVR (patient 32) and one with non-SVR (patient 34) were included. In addition, three anti-HCV negative plasma samples were tested against the six genotype 1 culture viruses. As positive control we used a previously tested chronic-phase genotype 1a serum sample, H06, shown to exhibit high levels of cross-neutralization *in vitro* against different genotypes [14]–[16], [38], and tested it against the DH6/JFH1 recombinant. Finally, to control for unspecific plasma inhibition or enhancement of infection, polyclonal IgG was purified from 100 µL plasma from three selected samples. IgG purification was performed using a Protein G HP Spin Trap/Ab Spin Trap and Ab Buffer kit (GE Healthcare) following manufacturer’s guidelines and quantified by standard methods (Department of Clinical Biochemistry, Copenhagen University Hospital, Hvidovre, Denmark). The purified IgG was tested against DH6/JFH1, DH6/JFH1_V157A, V787A, S905C, Q1247L_, DH1/JFH1, and DH5/JFH1 in 2-fold dilutions starting at 200 µg/mL.

### Statistics

Comparisons between NAb_50_-titers and treatment outcome and baseline data were done using Fisher’s exact test through SAS 9.2 software including SAS enterprise Guide. Differences between the viruses were tested using Wilcoxon’s signed rank test with a Bonferroni correction. Odds ratio (OR) and 95% confidence intervals (CI) used to evaluate potential predictors including gender, mode of infection, nationality, BMI, cirrhosis, age at treatment initiation, HCV RNA titer, and alanine aminotransferase (ALT), were manually calculated in Excel2000.

### Ethical Considerations

In concordance with Danish regulations, all participants provided their written and verbal informed consent to store their sample at the DANHEP biobank for later scientific use and the Danish Data Protection Agency (J nr 2009-41-4197), the National Health Research Ethics Committee of the Capital Region of Denmark (H-1-2009-145) approved the use of the samples in this study.

## Results

### Neutralizing Antibodies in Patients with Chronic Hepatitis C, Genotype 1, against Six Genotype 1 Recombinant Viruses in Correlation to Treatment Outcome

Thirty-nine patients with chronic hepatitis C, genotype 1, 23 with SVR and 16 with non-SVR, were enrolled in the study to determine their capability to neutralize three genotype-1a recombinant viruses (TN/JFH1, DH6/JFH1, and H77/JFH1) and three genotype-1b recombinant viruses (DH1/JFH1, DH5/JFH1, and J4/JFH1) [14], [16], [19].

The two patient groups were similar in terms of baseline variables (Table 1). All were treated with a weekly injection of pegylated interferon-α in combination with ribavirin. The mean duration of treatment was 49 weeks for the SVR group and 26.5 weeks for the non-SVR group. The reason for a significant shorter mean duration of treatment in the non-SVR group is termination of antiviral treatment if EVR is not achieved.

**Figure 1:**
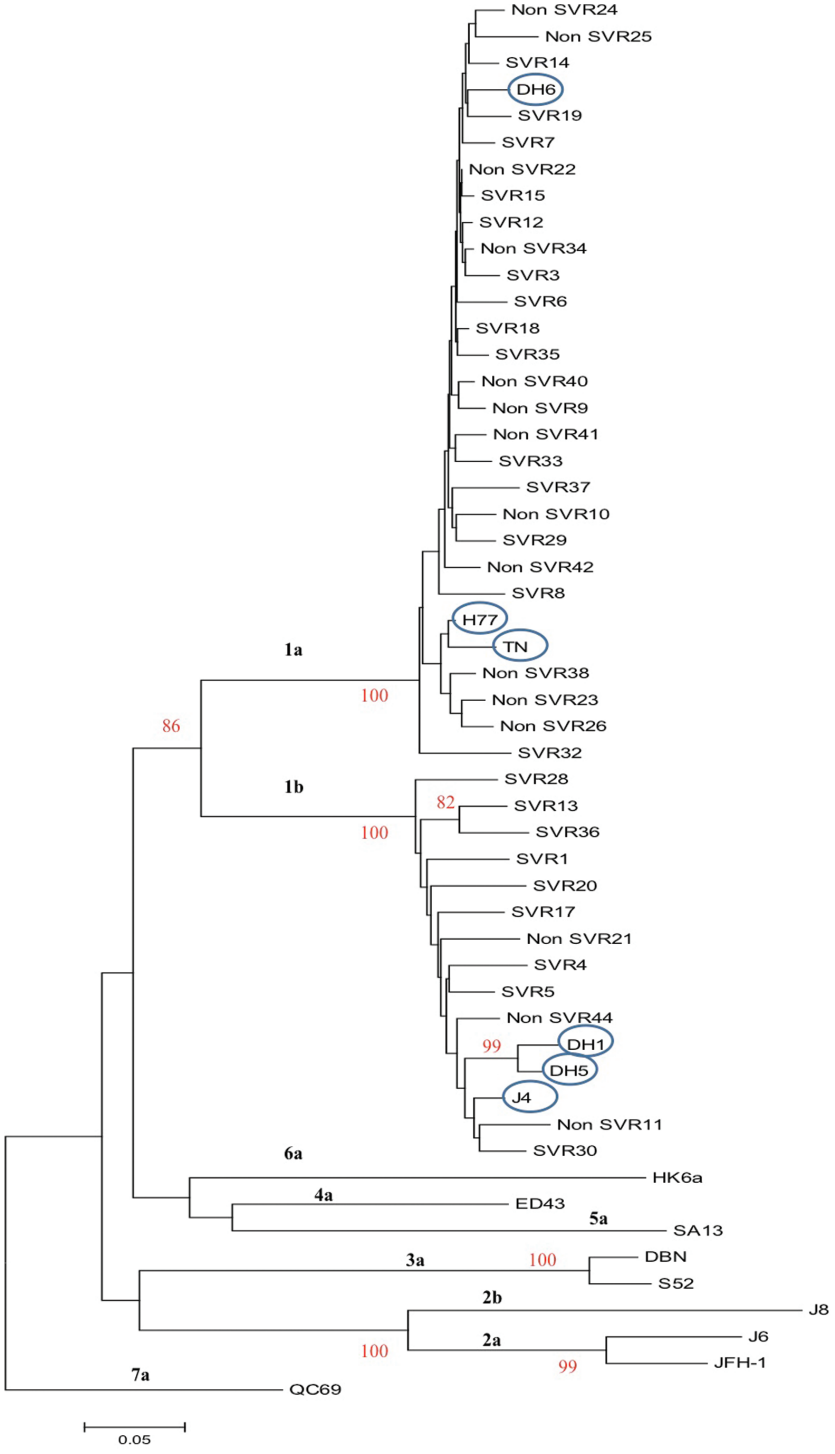
Phylogenetic analysis of the Core-E1 sequences of the HCV, genotype 1a and 1b, isolates from 38 patients. The analysis includes the Core-E1-sequences (nts. 868–1288; GenBank accession number AF009606) (one of the 39 included patients could not be subtyped and is not included in the tree), the corresponding sequence of the included genotype 1 culture viruses (circled) and culture viruses of other HCV genotypes. The QC69 (genotype 7a) is included as an out-group. The percentage of 1000 replicates in which the associated taxa cluster together in the bootstrap test is shown when >80%. The phylogenetic tree was constructed using the neighbor-joining method in the MEGA5 software. Each patient was assigned with a study number (also shown in Table 2) and the treatment outcome (SVR and non-SVR) is noted. The scale indicates the evolutionary rate of the branches.

During initial follow-up the HCV infected patients had not been subtyped; by Core-E1 sequence analysis [35], 26 and 12 patients were identified to be infected with HCV genotype 1a and 1b, respectively; one patient sample could not be subtyped (Figure 1).

**Figure 2:**
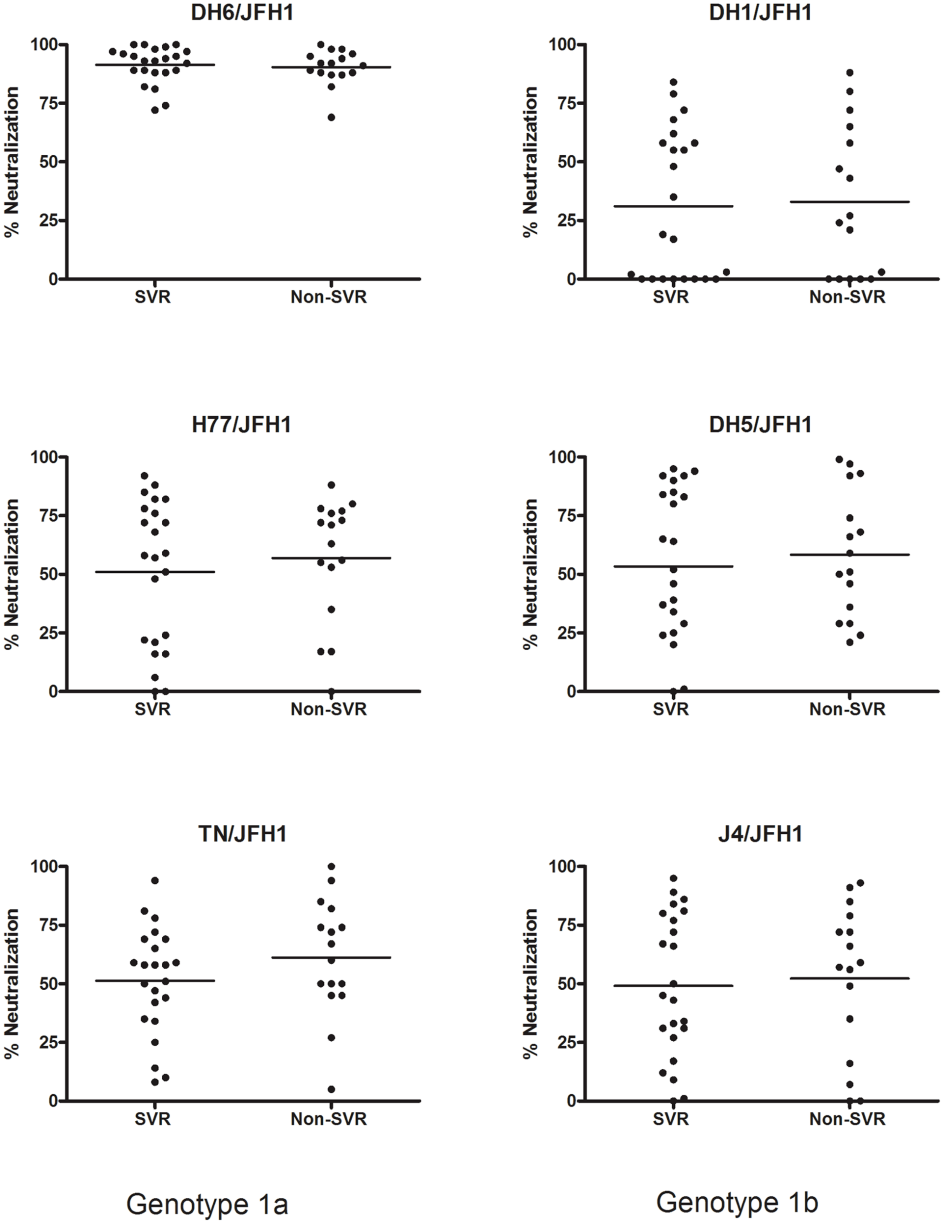
Percentage of neutralization against genotype 1a and 1b culture viruses in 39 patients with chronic HCV, genotype 1, infection. Plasma samples from 39 patients, taken prior to treatment initiation with pegylated interferon-α and ribavirin, were tested against genotype 1a Core-NS2 recombinants (TN/JFH1, DH6/JFH1, and H77/JFH1) and genotype 1b Core-NS2 recombinants (DH1/JFH1, DH5/JFH1, and J4/JFH1). The graph shows the percentage of neutralization in the samples at a 1∶50 dilution. Results are correlated with treatment outcome (SVR, Non-SVR) and each dot represents a patient. Dots located at the x-axis neutralized the culture virus ≤0%. For each group the mean is marked with a line.

**Figure 3:**
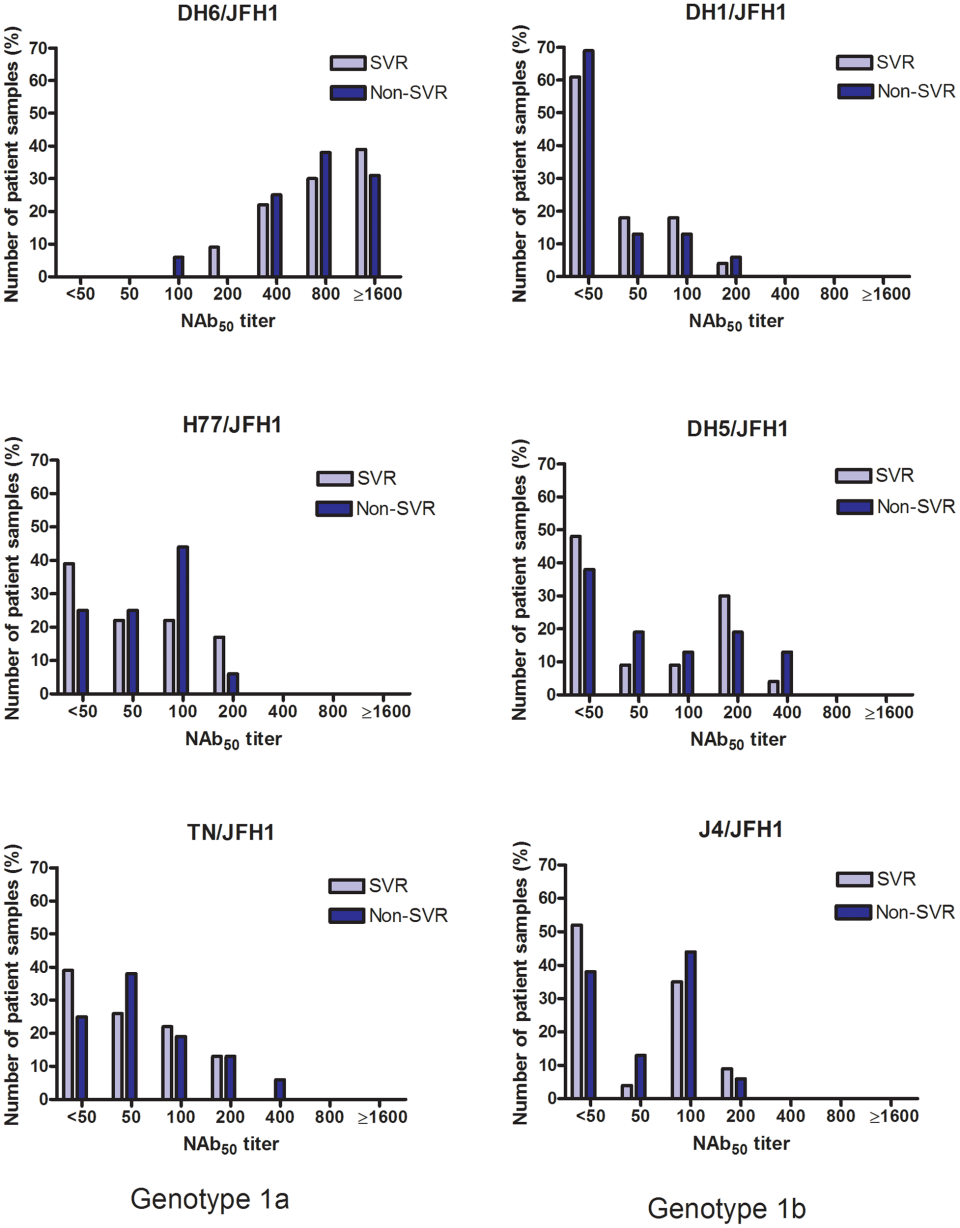
Neutralizing antibodies in 39 patients chronically infected with hepatitis C virus, genotype 1. Plasma samples from 39 patients, taken prior to treatment initiation with pegylated interferon-α and ribavirin, were tested against genotype 1a and 1b Core-NS2 recombinants in a two-fold dilution series starting from 1∶50. The graphs show the results for each virus. The NAb_50_-titers are the highest dilution where the patient samples were able to neutralize 50% of the virus. Each bar shows the percentage of total patients from each group (i.e. SVR (n = 23) and non-SVR (n = 16)) in relation to their NAb_50_-titer.

**Figure 4:**
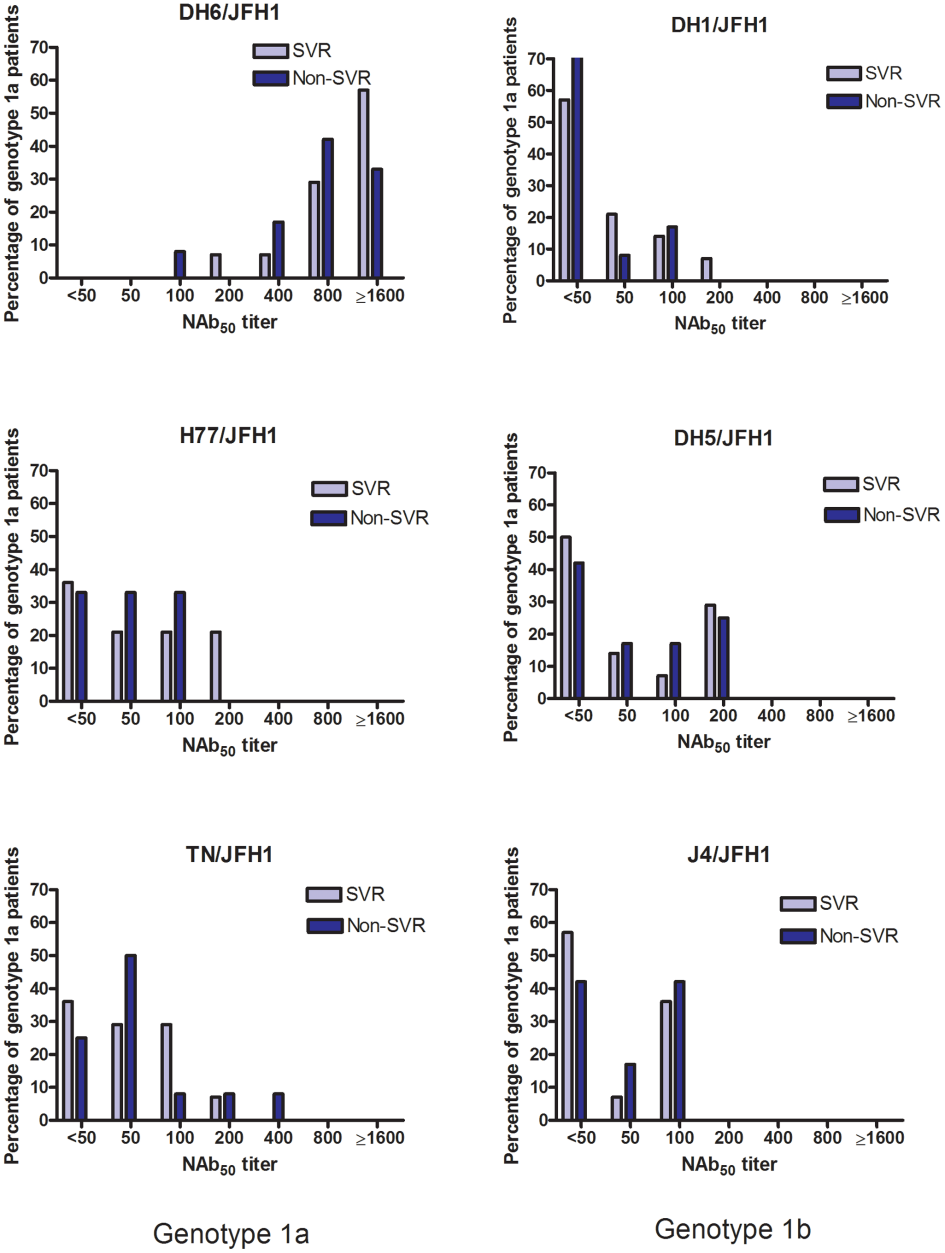
Neutralizing antibodies in 26 patients chronically infected with hepatitis C virus, genotype 1a. Plasma samples from 26 patients with chronic HCV infection, genotype 1a, taken prior to treatment initiation with pegylated interferon-α and ribavirin, were tested against genotype 1a and 1b Core-NS2 recombinants in a two-fold dilution series starting from 1∶50. The NAb_50_-titers are the highest dilution where the patient samples were able to neutralize 50% of the virus. Each bar shows the percentage of patients in the SVR (n = 14) and non-SVR (n = 12) group in relation to their NAb_50_-titer.

All patient plasma samples were tested in the *in vitro* neutralization assays at a 1∶50 dilution and grouped according to treatment outcome (Figure 2). The mean of the percentage of neutralization did not differ significantly between the two patient groups, however all samples showed a high percentage of neutralization against the DH6/JFH1 recombinant compared to the other culture viruses. This initial analysis was followed by dose-response analysis of the patient samples, to determine the highest dilution at which each virus infection was 50% neutralized (NAb_50_-titer). The results are shown in Table 2, ordered according to treatment outcome and HCV subtype. Correlating the NAb_50_-titers in the two groups (SVR vs. non-SVR) for each virus, showed no significant difference between the groups as calculated by Fischers exact test (Figure 3). Analyzing only genotype 1a patient samples, revealed no difference in NAb_50_-titers on a subtype specific level either (Figure 4). There were too few genotype 1b patients to make comparison in that group. Thus, the pre-treatment level of NAb did not correlate with treatment outcome.

**Table 2:** Neutralizing antibodies in 39 patients chronically infected with HCV, genotype 1, and treated with pegylated interferon/ribavirin.

			Genotype 1a			Genotype 1b		
Patient	Subtype	Outcome	DH6	H77	TN	J4	DH1	DH5
33	a	SVR	200	50	50	<50	<50	50
6	a	SVR	400	50	100	<50	<50	<50
12	a	SVR	800	50	<50	<50	<50	<50
14	a	SVR	800	<50	<50	<50	<50	<50
19	a	SVR	800	<50	100	<50	<50	50
32	a	SVR	800	100	100	50	<50	<50
3	a	SVR	1600	100	<50	100	50	200
7	a	SVR	1600	200	100	100	50	200
8	a	SVR	1600	200	50	100	50	100
15	a	SVR	1600	<50	<50	<50	<50	<50
18	a	SVR	1600	<50	<50	<50	<50	<50
37	a	SVR	1600	200	50	100	100	200
29	a	SVR	3200	<50	50	<50	100	<50
35	a	SVR	6400	100	200	100	200	200
17	b	SVR	200	<50	<50	<50	<50	<50
4	b	SVR	400	100	200	200	100	400
13	b	SVR	400	<50	<50	<50	<50	<50
28	b	SVR	400	<50	<50	<50	<50	<50
36	b	SVR	400	50	50	200	50	200
5	b	SVR	800	200	100	100	<50	200
20	b	SVR	800	50	<50	100	<50	100
30	b	SVR	800	<50	50	<50	<50	<50
1	b	SVR	1600	100	200	100	100	200
38	a	Non-SVR	100	100	50	50	<50	50
10	a	Non-SVR	400	100	100	100	<50	200
40	a	Non-SVR	400	50	50	<50	<50	50
22	a	Non-SVR	800	<50	<50	50	<50	<50
24	a	Non-SVR	800	50	50	100	<50	<50
25	a	Non-SVR	800	<50	50	<50	<50	<50
34	a	Non-SVR	800	<50	<50	<50	<50	<50
41	a	Non-SVR	800	50	50	100	50	100
9	a	Non-SVR	1600	100	200	100	100	200
42	a	Non-SVR	1600	100	400	100	100	100
23	a	Non-SVR	3200	<50	50	<50	<50	200
26	a	Non-SVR	3200	50	<50	<50	<50	<50
21	b	Non-SVR	400	100	100	100	<50	50
44	b	Non-SVR	800	100	100	200	50	400
11	b	Non-SVR	3200	100	200	100	400	400
43	ND	Non-SVR	400	200	<50	<50	<50	<50

NAb_50_-titer for each plasma sample against each genotype 1a and genotype 1b recombinant virus. The result is ordered according to treatment outcome, subtype and the NAb_50_-titer against DH6/JFH1 starting with the plasma sample with the lowest titer. NAb_50_-titer is the highest dilution where the plasma was able to neutralize 50% of the test culture virus.

**Figure 5:**
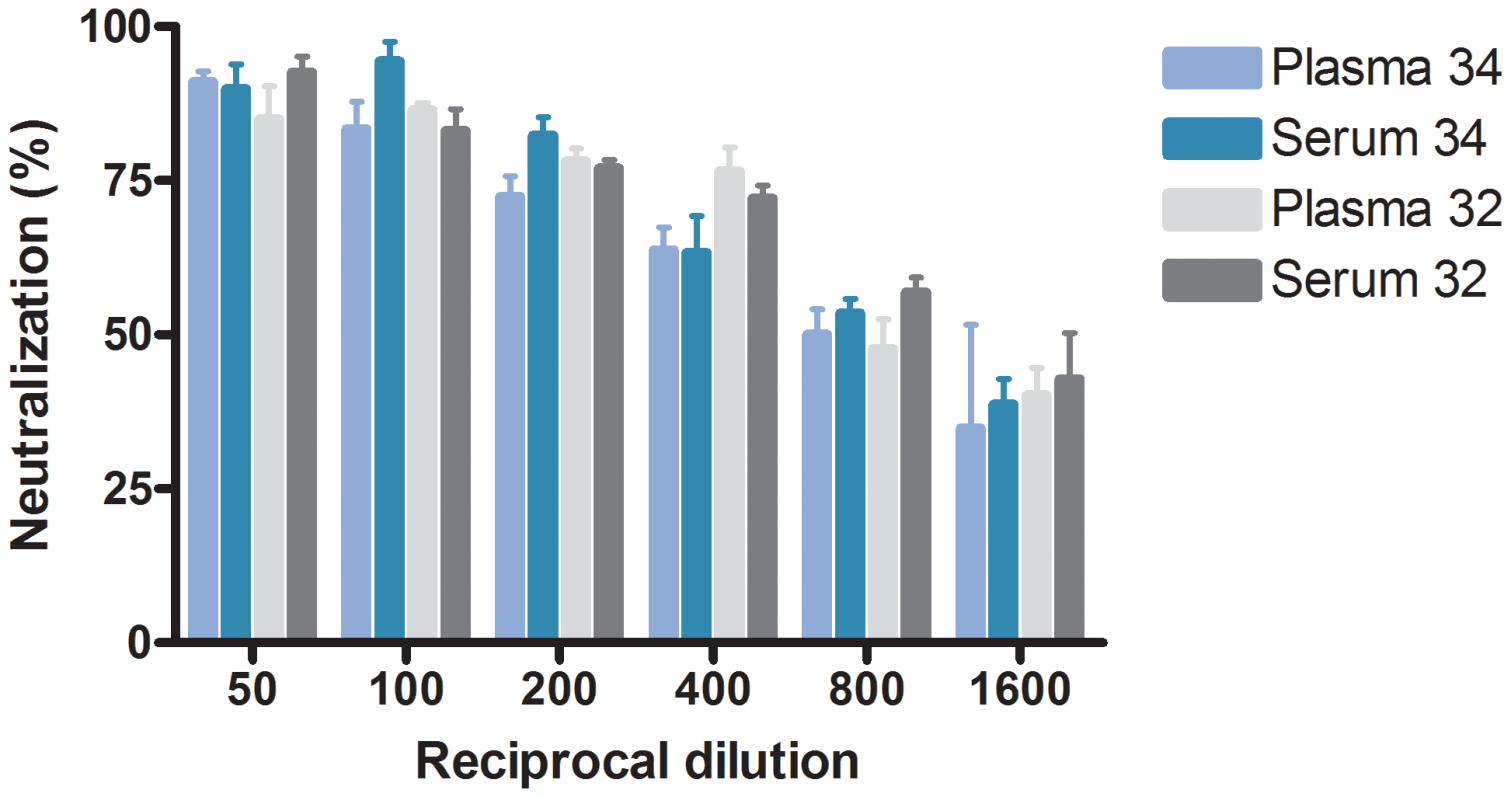
Testing the difference between plasma and serum in the neutralizing assay. The graph shows the reciprocal dilution on the x-axis and the percent neutralization on the y-axis. Each bar represents either plasma or serum from selected patients. Patient no. 32 is marked in gray scale and patient no. 34 is marked in blue scale colors. Error bars indicate SEM of triplicate determinations.

**Figure 6:**
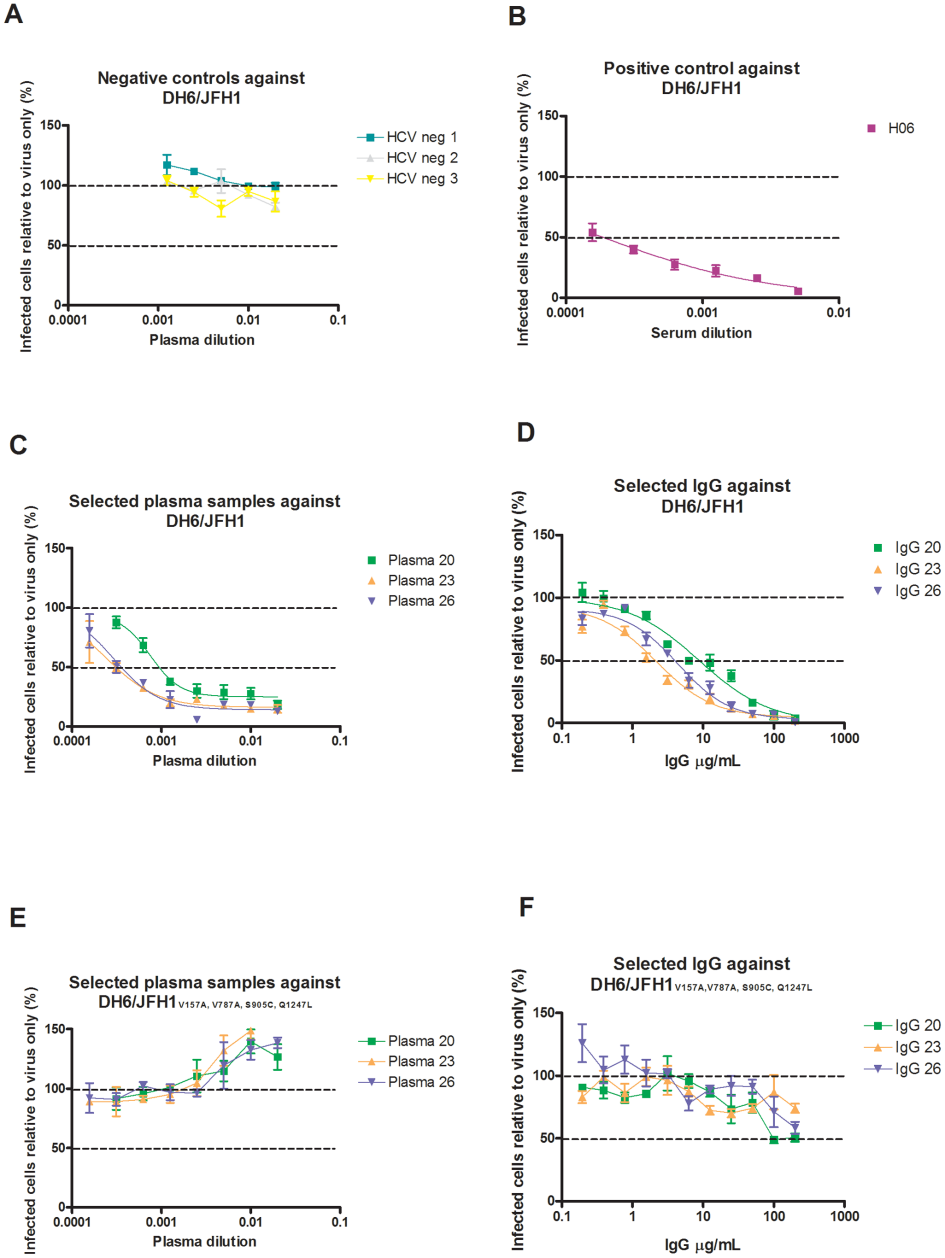
Neutralization susceptibility of DH6/JFH1. The figure illustrates the neutralization susceptibility of DH6/JFH1 with and without adaptive envelope protein mutations. Dotted lines indicate the infectivity at 50% and 100% compared to virus only. **A**, DH6/JFH1 against three HCV-negative plasma samples in 2-fold dilution starting from 1∶50. **B.** DH6/JFH1 against H06, a HCV positive serum sample, in a 2-fold dilution starting from 1∶200. **C.** DH6/JFH1 against 3 selected plasma samples in a 2-fold dilution starting from 1∶50. **D.** DH6/JFH1 against purified IgG from the same selected plasma samples in 2-fold dilution starting from 200 µg/mL. **E**. DH6/JFH1_ V157A, V787A, S905C, Q1247L_ against the same selected plasma samples in a 2-fold dilution starting from 1∶50. **F.** DH6/JFH1_ V157A, V787A, S905C, Q1247L_ against purified IgG from the same selected plasma samples in 2-fold dilution starting from 200 µg/mL. Error bars indicate SEM of triplicate determinations.

For control experiments, plasma and serum from the same patient at the same time point were tested against DH6/JFH1, and no significant difference was seen testing samples from two different patients (Figure 5). To control for unspecific neutralization, all the included 1a and 1b culture viruses were tested against three HCV negative plasma samples and no neutralization ≥50% was observed. Results are shown for DH6/JFH1 in Figure 6A. At the lowest dilution of 1∶50, a 2-fold infectivity enhancement was observed in DH1/JFH1, J4/JFH1, and H77/JFH1, which is a well- described phenomenon [39], [40]. In contrast, the previously tested chronic-phase serum, H06 was able to neutralize DH6/JFH1 with a NAb_50_ titer of 3200 (Figure 6B).

Finally, to examine specificity of neutralization DH6/JFH1, DH5/JFH1, and DH1/JFH1 were tested against purified IgG from selected samples with a high NAb_50_-titer against DH6/JFH1. Against DH6/JFH1 neutralization with plasma and IgG showed comparable curves, which indicate that neutralization was IgG mediated (Figure 6C and D). Of the 3 samples selected, 2 plasma samples (patient 20 and 23) neutralized DH5/JFH1 but not DH1/JFH1. Using purified IgG, neutralization was observed for DH5/JFH1 at concentrations of 200 and 100 µg/mL corresponding to the plasma neutralization results. Against DH1/JFH1, limited neutralization was observed at 200 µg/mL (∼50% neutralized), and in accordance with this result no neutralization was observed at 100 µg/mL. These results suggest that the neutralization was IgG-dependent, and that the enhancing factors in plasma could not mask significant neutralization.

### Importance of Adaptive Envelope Mutations for Increased Neutralization Susceptibility of the DH6/JFH1 Culture Virus

A significant difference in NAb_50_-titer was observed between DH6/JFH1 and the other included recombinant viruses (p<0.001). Unlike the other culture viruses, DH6/JFH1 contains two envelope mutations, I414T and Y444H, located in previously described neutralizing epitopes [41]–[43]. In order to address this difference in neutralization susceptibility, studies were performed using the DH6/JFH1_V157A, V787A, S905C, Q1247L_, without the envelope mutations. Neutralization assays were performed using both plasma and purified IgG from the three selected samples, and the results were compared to the DH6/JFH1 with envelope mutations (Figure 6C–F). None of the three samples with a reciprocal NAb_50_-titer of 800–3200 against the DH6/JFH1 could neutralize the DH6/JFH1_V157A, V787A, S905C, Q1247L_ ≥50% (Figure 6E). The results were the same for the purified IgG (Figure 6F). This clearly shows the importance of these adaptive envelope mutations for neutralization susceptibility, and could potentially explain the highly significant difference observed between this culture isolate and the other recombinants included in this study.

### Envelope Sequences of Patient Derived Viruses and Culture Viruses

We determined the E1E2 sequence of 16 patient derived viruses by direct sequencing. For all 16 patients we amplified the sequence from 3′end of Core to the 5′end of p7. All sequences, encoding the envelope proteins, were aligned with the corresponding sequences from the included culture viruses (Figure S2). An insertion in HVR1 was observed for patient 23 resulting in a histidine insertion (aa R384_T385insH) and a deletion was observed for patient 13 (aa575). Insertions in the HVR1 region have been described previously [44], [45], but deletion at position 575 has not been reported before. However, it is not a position that, to our knowledge, has previously been described to be important for neutralization.

**Figure 7:**
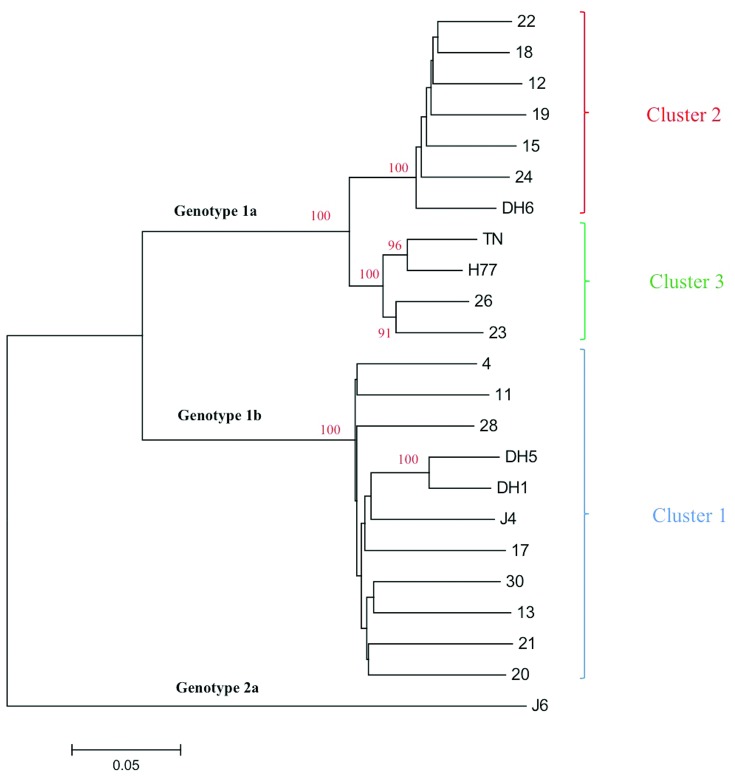
Phylogenetic analysis of the HCV E1E2 sequences of 16 patient derived viruses and 6 culture viruses. The analysis includes the E1E2-sequences (nts. 915–2579; GenBank accession number AF009606) of 16 genotype 1a and 1b patients and the corresponding sequence of the included genotype 1 culture viruses. J6 (genotype 2a) is included as an out-group. Phylogenetic analysis is otherwise performed as described in Figure 1 legend. The three major clusters are highlighted with different colors. Blue = genotype 1b, cluster 1, Red = genotype 1a, cluster 2, Green = genotype 1a, cluster 3.

**Figure 8:**
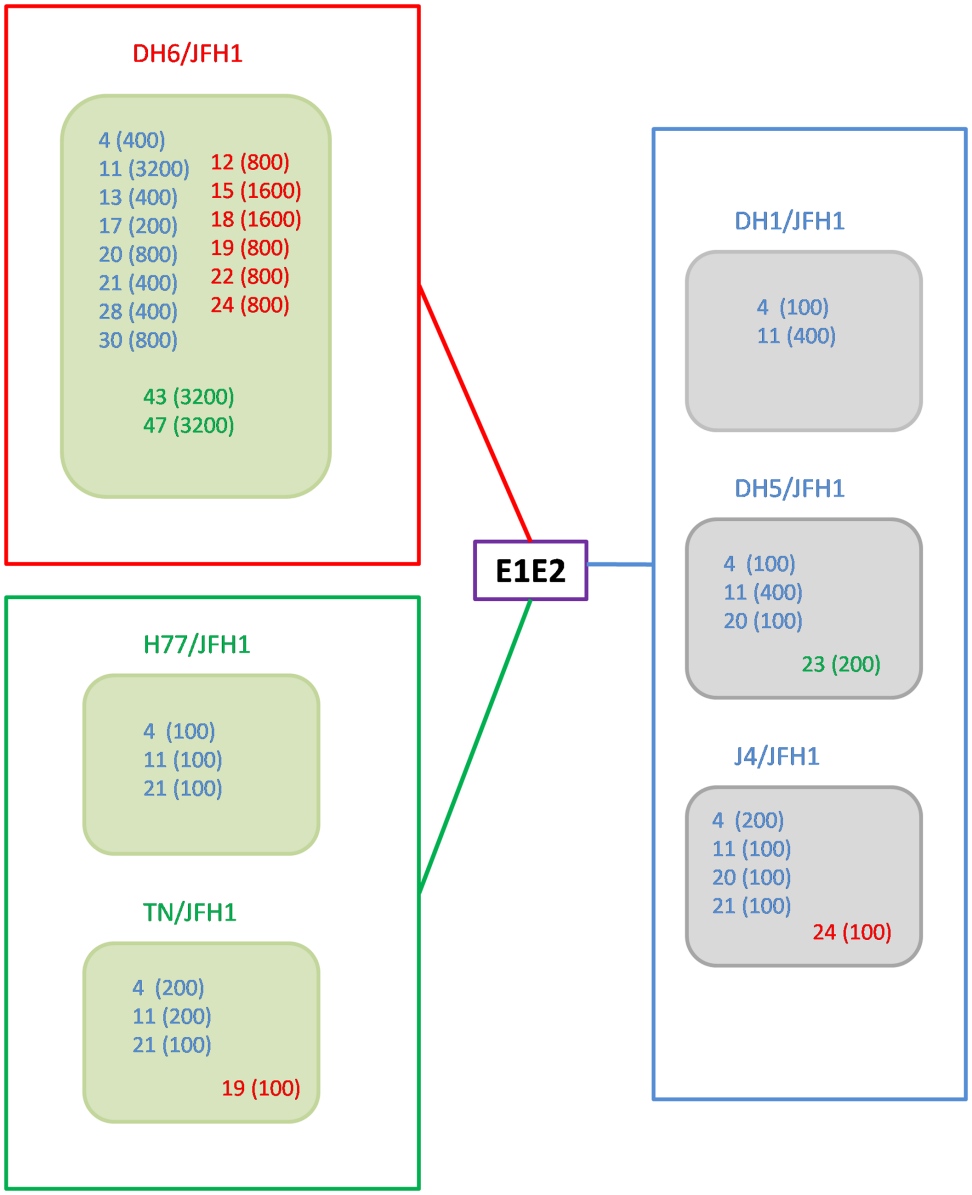
Phylogenetic relationship of the envelope sequence of the patient derived viruses and the culture viruses with associated NAb_50_-titer. 16 patient derived viruses and 6 culture viruses divided into three groups according to the sequence of the E1E2 analyzed in a phylogenetic tree (see Figure 7). The clusters are marked by different colors of the names of the samples and viruses, including a box framing the viruses in the cluster. Blue = genotype 1b, cluster 1; Red = genotype 1a, cluster 2; Green = genotype 1a, cluster 3. Only plasma samples with NAb_50_-titer ≥100 against genotype 1a viruses (green squares) and genotype 1b viruses (grey squares) are shown. The patient number is written in the squares and the titer against the virus is written in parentheses.

In order to be able to determine the genetic relationship between E1E2 of the included culture viruses and the patient derived viruses, the sequences were aligned and compared in a phylogenetic analysis (Figure 7). The genotype 1a and 1b clustered into two major groups. However, significant differences were also shown for the genotype 1a viruses, dividing them into two major genotype 1a clusters with DH6 in one cluster and H77 and TN in another. The major clusters are illustrated by different colors in Figure 7, and the clusters with the associated NAb_50_-titers are illustrated in Figure 8. There was no apparent link between relatedness of patient sequences with culture viruses used, and the corresponding neutralization results.

Taking a closer look at epitopes, previously highlighted as important for neutralization [42], [43], [46]–[50], the sequences of the patient derived viruses and the culture viruses did not show an obvious pattern connected to the neutralizing antibody titers (Figure S2). However, DH6 and TN viruses and two patient derived viruses (Patients 19 and 24) all have a histidine at position 444, which occurs naturally in the TN and the patients, but appear as an engineered adaptive mutation in the DH6 in combination with I414T. Of cluster 2 and 3 patient derived viruses, only plasma from patient 19 was able to neutralize TN with a NAb_50_-titer ≥100.

### Baseline Data and Neutralizing Antibodies

To determine if NAb_50_-titers were associated with other variables the mean geometric titer (NAb_50_GMT) were calculated and compared with baseline variables (Table 3). Patients with cirrhosis appear to have higher levels of NAb against all the included viruses than patients without, however, none were significant using Fisher’s exact test. Only 26 patients had a biopsy taken. The other baseline data do not show a consistent pattern. However, the results showed that patients with high BMI had significantly higher NAb_50_-titer against TN/JFH1 and DH5/JFH1 (p = 0.006 and 0.01), but only 33 patients had a BMI calculated. The viral load was not significantly associated with the NAb_50_-titer in this cohort.

**Table 3:** Neutralizing antibodies and the association with baseline data of 39 patients with chronic hepatitis C, genotype 1.

	DH6/JFH1	H77/JFH1	TN/JFH1	J4/JFH1	DH1/JFH1	DH5/JFH1
Male	982	65	60	51	42	63
Female	635	47	53	56	33	75
Intraveneous drug users	841	64	67	58	39	61
Non-IDU/unknown	869	56	53	50	39	70
BMI >25*	800	67	67	61	39	92
BMI ≤25*	993	57	50	48	39	48
Cirrhosis no**	768	52	61	48	35	59
Cirrhosis yes**	1089	86	86	79	50	147
Age ≤45	907	60	60	47	47	57
Age >45	841	58	57	55	36	71
HCV RNA ≥600.000	822	56	60	51	38	69
HCV RNA <600.000	939	65	56	53	40	62
ALT ≥70/100	678	50	59	50	36	61
ALT <70/100	1131	71	56	56	43	73

Geometric means of the NAb_50_-titer of the different viruses compared to baseline variables.

* Only 33 patients had a BMI calculated.

** Only 26 patient had a biopsy taken.

## Discussion

We tested a well-defined patient group, chronically infected with HCV, genotype 1, against six genotype 1 recombinant cell-culture viruses. Using this novel approach of testing plasma samples against an entire panel of genotype 1 culture viruses, we were able to determine the level of NAb against several different viruses, in patients with a positive or negative treatment outcome. We found that the pre-treatment level of NAb against HCV genotype 1 recombinant viruses was not a predictor of PEGIFN/RBV treatment outcome in patients with chronic HCV, genotype 1, infection. The panel of viruses, consisting of three genotype 1a and three genotype 1b recombinant viruses, enabled analyzes of the levels of neutralization in patients with a HCV genotype 1a, infection, thus studying subtype-specific neutralization. However, no correlations between NAb_50_-titers in regard to treatment outcome on a subtype specific level were found.

Our findings correspond to the results previously reported by Boo et al. [31] who did not find a difference between 17 patients infected with HCV of different genotypes and treatment outcome when tested against one genotype 1a virus, pE1E2H77c in a HCVpp system. However, we tested 39 patients, all infected with genotype 1, against a panel of genotype 1 recombinant culture viruses, and still did not find any genotype or subtype differences in the two groups. This strengthens the findings, that there is no difference in NAb in patients with SVR compared to patients with non-SVR, tested against recombinant viruses of the same genotype. In contrast, Saseyema et al. [32] found that high NAb_50_-titers against the genotype 2a J6/JFH1 virus could predict SVR, among a total of 65 patients with genotype 1b, thereby testing cross-neutralizing antibodies. We did not test cross-neutralizing antibodies against different genotypes, but against different subtypes and did not find the same correlation.

A drawback in the design of the present study is that we only tested prototype culture viruses against the patient plasma antibodies, and thus could not detect whether there is a NAb response against the patients’ own viral population at the given time. However, it has previously been shown that a NAb response fails to efficiently neutralize quasispecies, dominant at the given time point, but shows significant neutralization against quasispecies from a previous time point [29]. This decreases the likelihood of seeing an efficient NAb response against the patients’ own quasispecies. Furthermore, setting out to add an additional predictor for treatment outcome in a clinical setting, a representative virus panel would be needed to test incoming patient samples. The E1E2 sequence of the culture viruses used in this study represented a broad spectrum of genotype 1a and 1b E1E2 sequences from the Los Alamos database when compared in a phylogenetic analysis. Two major clusters were seen among 47 genotype 1a sequences, where H77 and TN were represented in one, and DH6 were represented in another. For genotype 1b sequences, the same phenomenon was observed showing two clusters. However, the three 1b strains, included in our study, were only represented in one cluster, which were the major of the two. To counter the possible differences between the prototype strains and the patient samples, the entire E1E2 sequences were determined for selected patient samples. In a phylogenetic analysis, the genotype 1b patient samples cluster together and the genotype 1a samples divide into the two clusters with DH6, and H77 and TN, respectively. The major part of the genotype 1a patient derived viruses cluster with DH6. This is probably due to the origin of viruses used for developing these culture system, where DH6 culture viruses was developed from a Danish patient [19], whereas both H77 and TN come from U.S strains [51], [52]. When comparing the amino acids at positions, previously highlighted as important for neutralization, we found, to our knowledge, no major differences which could be linked to the neutralization. At position 444, both TN and two patient samples had a histidine, which was added as an engineered adaptive mutation in the highly neutralization susceptible DH6. However, only one of the two samples was able to neutralize the TN, suggesting the histidine plays a minor role in the neutralization of this culture virus. The close genetic relationship between the culture viruses and the patient derived viruses, in phylogenetic analysis and in known neutralizing epitopes, suggests that the prototypes used are suitable as surrogates for the patients’ own viruses in this setting.

A novel finding of the study was the considerable difference in NAb_50_-titers between the different recombinant viruses, even those of the same subtype. All patient samples showed a significantly higher NAb_50_-titer against DH6/JFH1 when compared to the other genotype 1 isolates. In contrast to the other included viruses, the DH6/JFH1 has two engineered adaptive mutations in epitopes highly relevant for neutralization. The I414T mutation is placed in epitope I, a genomic area which has been known for its potential as neutralizing target relevant for future vaccine development [41]–[43], [46], [48]. The Y444H is on the other hand positioned in epitope II, which previously was shown to be a target for non-neutralizing antibodies, but recent studies show efficient neutralization with antibodies targeting this epitope [41], [49]. Subsequent analysis presented in this study, testing three selected patient plasma samples and extracted IgG against the DH6/JFH1_V157A, V787A, S905C, Q1247L_without envelope mutations showed lack of neutralization, supporting the importance of these adaptive envelope mutations for neutralization susceptibility. Generally, this finding underlines the importance of testing multiple strains in neutralization studies, as cell culture viruses reflect quasispecies, developed in an *in vitro* setting without the pressure from the immune system.

Another shortcoming of our study is the low number of patients included. However, the difference between the NAb_50_-titer in the two groups is so small that, even if we had the chance to include additional patients, the likelihood of revealing a difference would be limited. Testing NAb of 39 patients against six different culture-adaptive viruses is a considerable effort, and this study is strengthened by the fact that for all viruses, including the highly neutralizable DH6/JFH1 with the envelope mutations, we observed no correlation between NAb_50_-titer and treatment outcome.

Lastly, we made an analysis on the baseline data and the NAb_50_-titer, which revealed that even though the NAb_50_-titers did not differ in relation to treatment outcome, an association could exist between cirrhosis and high levels of NAb. Such association would support the knowledge that increasing time of exposure increases the risk of developing cirrhosis and increases the level of neutralizing antibodies [25], [33], [53], [54]. Surprisingly, we found a significant association between the NAb_50_-titers against two viruses, TN/JFH1 and DH5/JFH1 and the BMI of the patients, which has to our knowledge not previously been shown in patients with chronic HCV infection. These add-on analyses could, however, represent merely random variation, and given that the neutralization titers against these viruses were generally low, further studies would be required to make a clear association. It is worth mentioning that the viral load was not associated with the NAb_50_-titer in our cohort.

In conclusion, we tested plasma samples from patients, treated for chronic HCV, genotype 1, infection, for potential neutralization ability in relation to treatment outcome. No significant difference in NAb_50_-titers in patients with SVR versus non-SVR was identified. Nevertheless, this study is the first to test the level of NAb in patients infected with HCV, genotype 1, against a panel of genotype 1a and 1b recombinant viruses. Interestingly, the presented results in addition revealed the importance of culture adaptive mutations with respect to neutralization susceptibility, which emphasizes the importance of using multiple culture viruses in neutralization studies in HCVcc-systems.

## Supporting Information

Figure S1 **E1E2 alignment of the amino acid sequence of 6 culture viruses used.** The names of the isolates are listed at the left. The beginning of the proteins is marked with the aa positions according to H77 (GenBank accession number AF009606) as is the positions with engineered adaptive mutations (position 414 (DH6), 444 (DH6) and 734 (DH5)). Variable residue positions are marked in grey.
(PDF)

Figure S2 **E1E2 alignment of the amino acid sequence of 16 patient derived viruses and 6 culture viruses used.** The names of the isolates and the patient numbers are listed at the left and variable residue positions are marked in grey. Positions of previously reported epitopes shown to be important for neutralization are marked with a square; Purple squares mark the glycosylation sites [47], Green, blue, and red squares mark sites found to be binding residues for lead human monoclonal antibodies [42], [46], [48], orange squares marks epitope II [49] and yellow squares mark three positions found to be important for cell entry [50]. Positions may be mentioned in several studies**.** The aa position is listed above according to H77 (GenBank accession number AF009606).
(PDF)
