# Supplementary figures and images for: Neutralizing Antibodies in Patients with Chronic Hepatitis C, Genotype 1, against a Panel of Genotype 1 Culture Viruses: Lack of Correlation to Treatment Outcome
Source: PLoS One. 2013 May 7;8(5):e62674. doi: 10.1371/journal.pone.0062674 (PMC3646876; doi:10.1371/journal.pone.0062674)

Figure S1

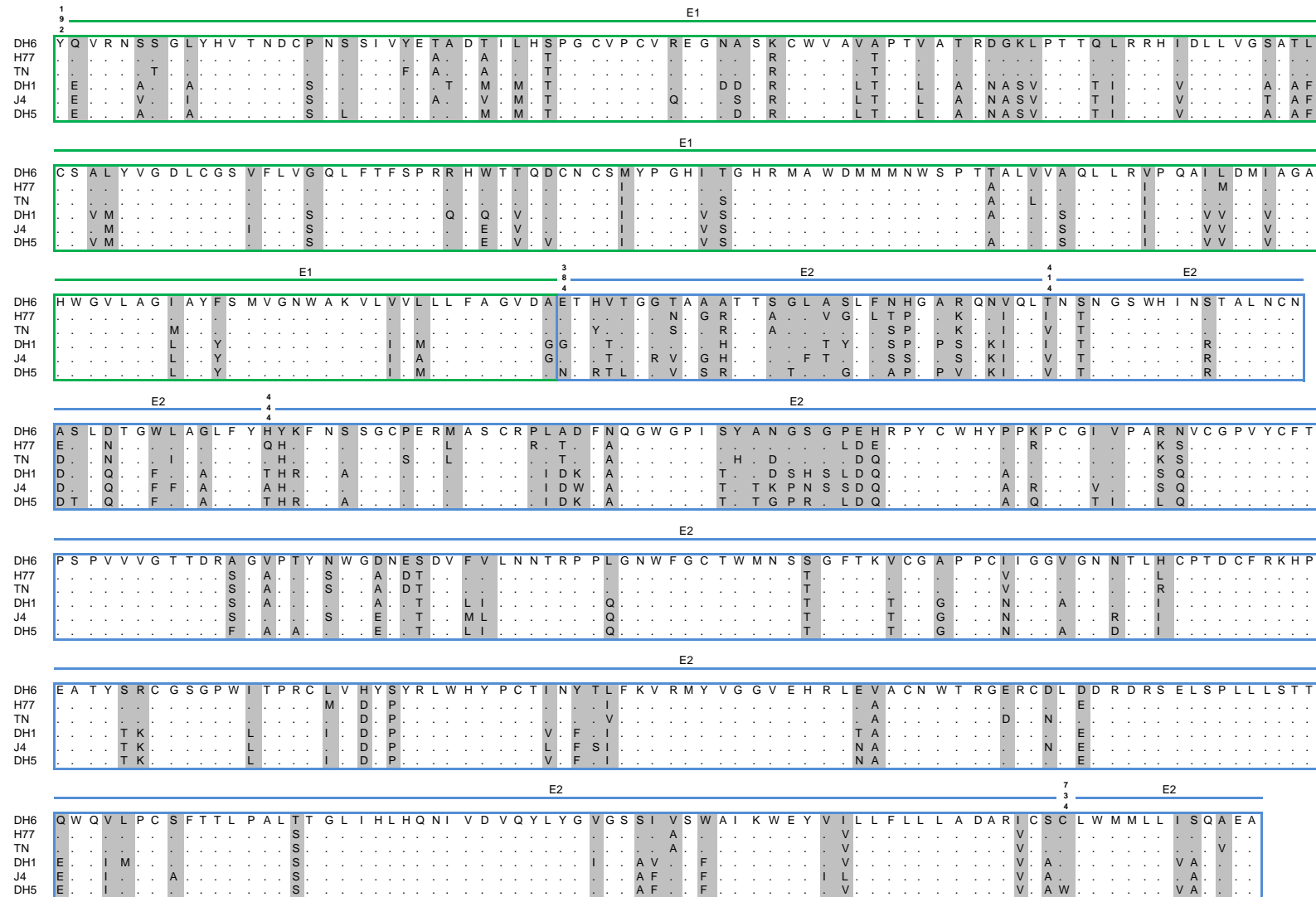

Supplement: Figure S1 — E1E2 alignment of the amino acid sequence of 6 culture viruses used. The names of the isolates are listed at the left. The beginning of the proteins is marked with the aa positions according to H77 (GenBank accession number AF009606) as is the positions with engineered adaptive mutations (position 414 (DH6), 444 (DH6) and 734 (DH5)). Variable residue positions are marked in grey. (PDF) [file pone.0062674.s001.pdf]

Figure S2 1/4

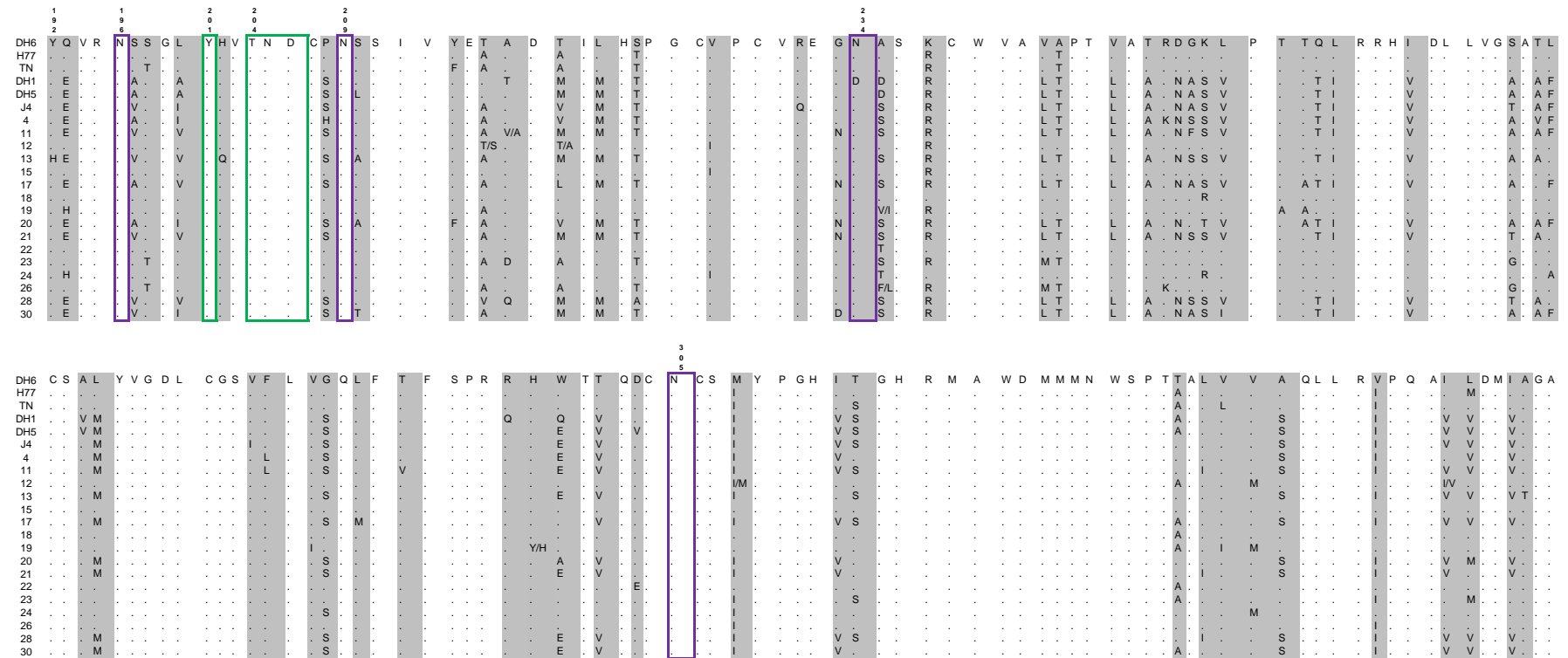

2/4

[illegible]

Figure S2 3/4

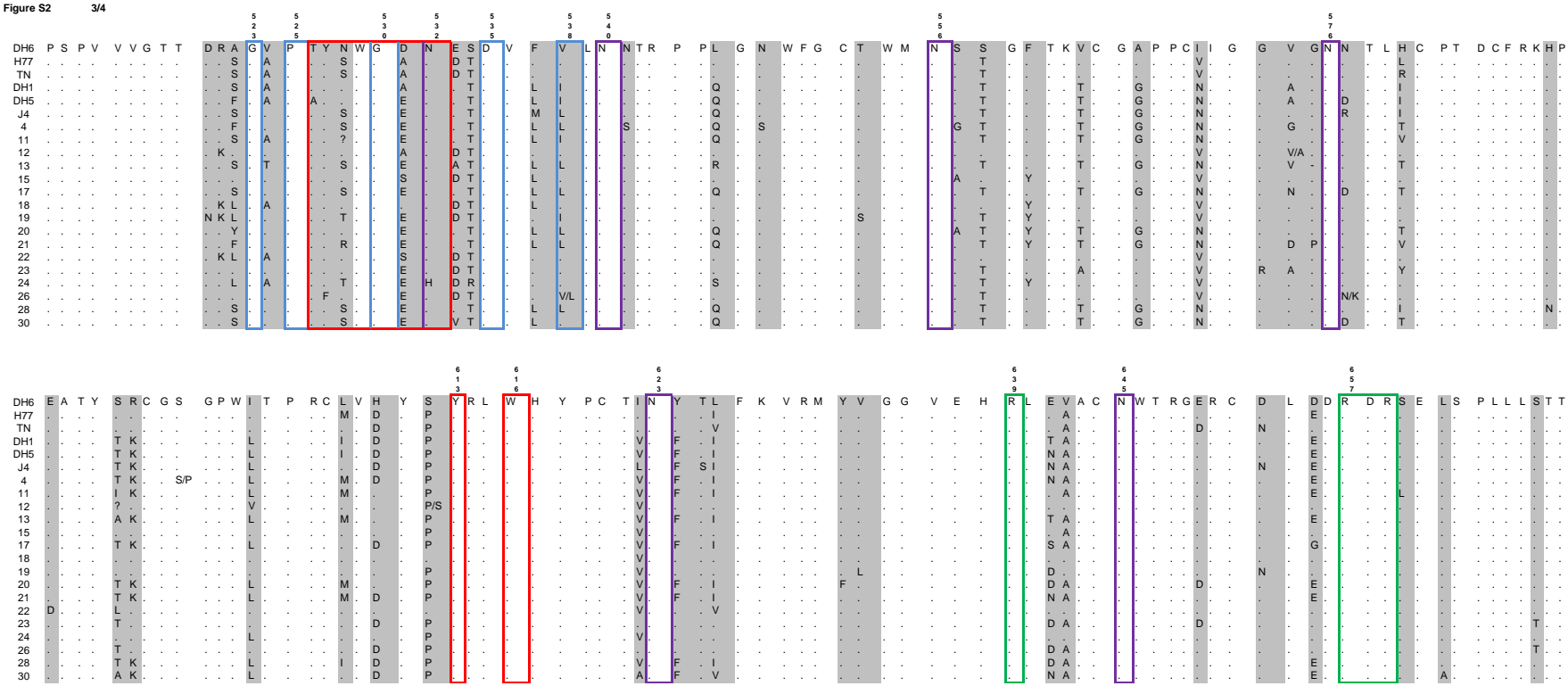

**4/4**

[illegible]

Supplement: Figure S2 — E1E2 alignment of the amino acid sequence of 16 patient derived viruses and 6 culture viruses used. The names of the isolates and the patient numbers are listed at the left and variable residue positions are marked in grey. Positions of previously reported epitopes shown to be important for neutralization are marked with a square; Purple squares mark the glycosylation sites [47], Green, blue, and red squares mark sites found to be binding residues for lead human monoclonal antibodies [42], [46], [48], orange squares marks epitope II [49] and yellow squares mark three positions found to be important for cell entry [50]. Positions may be mentioned in several studies. The aa position is listed above according to H77 (GenBank accession number AF009606). (PDF) [file pone.0062674.s002.pdf]
